# Thermosensitive and mucoadhesive gels containing solid lipid nanoparticles loaded with fluconazole and niosomes loaded with clindamycin for the treatment of periodontal diseases: a laboratory experiment

**DOI:** 10.1186/s12903-024-04322-6

**Published:** 2024-05-11

**Authors:** Zahra Saeidi, Rashin Giti, Azadeh Emami, Mehdi Rostami, Farhad Mohammadi

**Affiliations:** 1 Department of Pharmaceutics, Faculty of Pharmacy, Shahid Sadoughi University of Medical Sciences and Healthcare Services, Yazd, Iran; 2 Department of Prosthodontics, Faculty of Dentistry, Shiraz University of Medical Sciences, Shiraz, Iran

**Keywords:** Clindamycin, Fluconazole, Niosomes, Periodontal disease, SLNs

## Abstract

**Background:**

Periodontal diseases may benefit more from topical treatments with nanoparticles rather than systemic treatments due to advantages such as higher stability and controlled release profile. This study investigated the preparation and characterization of thermosensitive gel formulations containing clindamycin-loaded niosomes and solid lipid nanoparticles (SLNs) loaded with fluconazole (FLZ), as well as their in vitro antibacterial and antifungal effects in the treatment of common microorganisms that cause periodontal diseases.

**Methods:**

This study loaded niosomes and SLNs with clindamycin and FLZ, respectively, and assessed their loading efficiency, particle size, and zeta potential. The particles were characterized using a variety of methods such as differential scanning calorimetry (DSC), dynamic light scattering (DLS), and Transmission Electron Microscopy (TEM). Thermosensitive gels were formulated by combining these particles and their viscosity, gelation temperature, *in-vitro* release profile, as well as antibacterial and antifungal effects were evaluated.

**Results:**

Both types of these nanoparticles were found to be spherical (TEM) with a mean particle size of 243.03 nm in niosomes and 171.97 nm in SLNs (DLS), and respective zeta potentials of -23.3 and -15. The loading rate was 98% in niosomes and 51% in SLNs. The release profiles of niosomal formulations were slower than those of the SLNs. Both formulations allowed the release of the drug by first-order kinetic. Additionally, the gel formulation presented a slower release of both drugs compared to niosomes and SLNs suspensions.

**Conclusion:**

Thermosensitive gels containing clindamycin-loaded niosomes and/or FLZ-SLNs were found to effectively fight the periodontitis-causing bacteria and fungi.

## Introduction

Periodontal diseases refer to the inflammatory destruction of the gingiva, cementum, periodontal ligament, and alveolar bone. Gingivitis and periodontitis are two kinds of periodontal disease. Gingivitis is a reversible inflammation of the gingiva, characterized by symptoms like redness, bleeding, and swelling. Meanwhile, the spread of gingival inflammation to the periodontal ligament, cementum, and alveolar bone can lead to irreversible periodontitis. Periodontitis affects a significant proportion of the global adult population, with the mildest (50%) and the most severe forms (9.8%). Failure to treat this disease can lead to a range of negative consequences including tooth loss, lower self-esteem, altered speech, and diminished quality of life. Moreover, periodontitis can pose a risk to general health, as it is a risk factor for cardiovascular disease, diabetes, hypertension, respiratory illness, and myocardial and cerebral infarction [1–3]. Several factors are associated with an increased risk of periodontal disease, including age, smoking, and diabetes, as well as systemic factors such as medication and hormones, genetic factors, disorders of the immune system, and a history of periodontal surgery [2, 4].

Treatment of periodontal diseases involves local or systemic administration of antimicrobial agents. Given the potential drawbacks of systemic antibiotics such as side effects, bacterial resistance, poor distribution, low selectivity, and burst release, local delivery of antibiotics has emerged as a preferred alternative. Due to the absence of a topical formulation with these characteristics in the clinical practice, this approach offers several advantages including high drug concentration at the infection site, extended drug release, minimal side effects, and targeted drug delivery [5]. Furthermore, the periodontal pocket is easily accessible from the oral cavity. Recently, nanoparticles ranging from 10 to 1000 nm, capable of entrapping drugs internally, have been investigated for their potential in periodontal disease treatment. This is due to their advantages, including prolonged drug release profiles, targeted delivery to specific organs, and high drug loadings [6–8].

Niosomes are multilamellar vesicles composed of an amphiphilic component and a nonionic surfactant surrounding an aqueous core, which can carry both hydrophilic and hydrophobic drugs. Nonionic surfactants are preferred to cationic and anionic surfactants, because they are less irritable, while biodegradable, biocompatible, and non-immunogenic. These nanoparticles can be administered via different routes including oral, parenteral, and topical. The noisome stability facilitates their storage and handling [9].

Solid lipid nanoparticles (SLNs) are composed of 0.1–30% w/w solid lipid dispersed in an aqueous medium and 0.5–5% w/w surfactant as a stabilizer if necessary. These nanoparticles offer several benefits including a controlled release profile, and utilization of nontoxic and biodegradable lipids, besides improving the stability of a compound against light, oxidation, and hydrolysis [10].

Thermosensitive polymers, especially those with mucoadhesive properties, such as poloxamers, chitosan, and sodium alginate can undergo a phase transition from liquid to gel in response to an increase in temperature (around body temperature). Some researches improve advantages of these local formulations such as precise drug targeting, high absorption, and increased therapeutic efficacy due to prolonged residence time [5, 11, 12]. Poloxamer 407 (commercially known as Pluronic®) is a triblock polymer composed of a hydrophobic center (polypropylene glycol unit) flanked by two hydrophilic terminals (polyethylene glycol units). Thermosensitive gelation can be achieved at a certain concentration (above 15%). Meanwhile, the gelation temperature is concentration-dependent; that is, higher concentrations of thermosensitive polymer lead to lower gelation temperatures [13, 14].

Clindamycin and FLZ are rather small molecules with molecular weight 461.4 g/mol and 306.27 g/mol respectively. So, it seems they can kind of cross through epithelium of oral cavity. Clindamycin is an antibiotic with a wide range of action against gram-positive and aerobic microorganisms by binding to the 50 s subunit of the bacterial ribosome and inhibiting protein synthesis. Although there is no previous study evaluating local absorption of clindamycin and fluconazole in oral cavity directly, even if a small amount of them is absorbed from the oral cavity, they will be swallowed after administration finally and could absorb in duodenum. In addition, it possesses unique pharmacological properties, such as the ability to reduce the adhesion of bacteria to mucosal surface epithelial cells. Systemic administration of clindamycin can cause its short half-life and adverse effects like nausea, abdominal pain, diarrhea, and increased risk of clostridium difficile [15].

Recent studies have shown that yeasts can coexist with bacterial infections in periodontal disease and exhibit a synergistic relationship with them. For instance, *Candida albicans* has been found to enhance the viability of β-hemolytic streptococci [16]. FLZ is an antifungal drug that inhibits the production of ergosterol in the fungal membrane. Due to its systemic side effects such as diarrhea, vomiting, stomach upset, and rush, there is a growing interest in developing a local formulation of FLZ [17]. The present study was designed to investigate a local dosage form comprising a thermosensitive gel containing clindamycin niosomes and FLZ solid lipid nanoparticles as a potential applicable dosage form that release these drugs in extended profiles for the treatment of periodontal diseases.

## Materials and methods

### Materials

FLZ and clindamycin were respectively supplied by Zahravi and Sepidaj pharmaceutical companies (Iran). Pluronic 407® and chloroform were purchased from Bio Basic (Canada) and Merck (Germany), respectively. All other chemicals were of analytical grade.

### Methods

#### Fourier transform infrared spectroscopy (FT-IR)

FT-IR spectra of pure clindamycin and FLZ were recorded at room temperature and wavelength ranging from 450 to 4000 cm^−1^ (FTIR; Perkin Elmer, USA).

#### Calibration curve of clindamycin and FLZ

Standard concentrations of clindamycin hydrochloride and FLZ were prepared by serial dilution in water. UV spectrophotometer (Unico, USA) was used to measure the ultraviolet (UV) absorbance of clindamycin hydrochloride (at 225 nm) and FLZ (253 nm), and calibration curves were plotted.

#### Preparation of clindamycin-loaded niosomes

Niosomes were prepared using the thin film hydration method, where the mixture of cholesterol and tween 80 at a molar ratio of 1:19 was dissolved in chloroform and then placed in a rotary vacuum evaporator (IKA; Germany) at 60 °C for 30 min to remove chloroform under reduced pressure. The resulting thin film was kept in a desiccator for 24 h to ensure the complete removal of chloroform. The thin film was hydrated with phosphate buffer saline (PBS) (pH = 7.4) containing dissolved clindamycin at a rotation speed of 60 rpm at room temperature for 30 min. Then, the mixture was sonicated for 20 min in a bath sonicator (Elma; Germany) and stored in the refrigerator for further evaluations [18, 19].

#### Preparation of FLZ-loaded SLNs

SLNs were prepared using the high shear homogenizing method. The formulation was comprised of stearic acid and FLZ with the molar ratio of 2:1. Initially, stearic acid was melted at 75 °C, and then, FLZ was slowly added until completely dissolved, resulting in the lipid-drug mixture. The aqueous phase, containing 1% tween 80, was heated to the same temperature and lipid-drug mixture was added dropwise to the aqueous phase while stirring at 10,000 rpm using a magnetic stirrer (Heidolph; Germany). The resulting mixture was cooled to room temperature, sonicated for 20 min, and stored in the refrigerator for further tests [20].

#### Differential scanning calorimetry (DSC)

Thermal properties of FLZ-SLNs, unloaded SLNs, pure FLZ, stearic acid, tween 80, clindamycin-loaded niosomes, unloaded niosomes, pure clindamycin, and cholesterol were scanned with a heating rate of 10 C°/min between 25–200 °C by DSC (Sanaf Electronics, Iran).

#### Entrapment efficacy (EE)

The two formulations were centrifuged (Sigma 2016kl; Germany) at 13,000 rpm for 90 min at 4 °C to separate free drug from the drug-loaded noisome and FLZ-SLNs. The clear supernatants were then separated and analyzed using a UV spectrophotometer at 225 and 253 nm, respectively. The percentage of drug entrapment efficiency (EE %) was calculated using the following equation:

$$EE\%=\lbrack total\;drug-drug\;in\;supernatant/total\;drug\rbrack\times100$$

#### Particle size and zeta potential

Mean diameter of niosomes and SLNs, as well as zeta potential at 25 °C were determined using Dynamic Light Scattering (DLS) (Zetasizer; Malvern, UK).

#### Transmission electron microscopy (TEM)

The shape of FLZ-SLNs, unloaded SLNs, clindamycin loaded niosomes, and unloaded niosomes was determined using a transmission electron microscope (Zeiss; USA). A drop of each sample was placed on the surface of a carbon coated copper grid and allowed to dry for 10 min prior to examination.

#### In-vitro drug release of FLZ-SLNs and clindamycin-loaded niosomes

The in-vitro drug release of each formulation was evaluated using a dialysis sac (cut-off = 12,000 Da). Two mL of each formulation was transferred to the sac and placed in 100 mL PBS at pH = 7.4. The sac was stirred on a heater stirrer (Heidolph; Germany) at 100 rpm and 37 °C. Equal volume samples were withdrawn at predetermined intervals and replaced with fresh PBS. The concentration of clindamycin and FLZ were measured using UV spectrophotometer.

#### Preparation of clindamycin-loaded noisome and FLZ-loaded SLNs thermosensitive gel

18% w/v of Pluronic®407 was slowly added to the clindamycin-loaded noisome and FLZ-loaded SLNs at 5 °C while stirring until completely dissolved, and then refrigerated for further tests [13, 14].

#### Gelling temperature measurement

After adding 18% w/v Pluronic®407 to the clindamycin-loaded noisomes and FLZ-loaded SLNs, the mixture was heated gradually from room temperature (25 °C) using a heater stirrer with magnetic stirring while monitoring the temperature using a thermometer. The temperature at which the magnetic stirring ceased was recorder.

#### Viscosity measurement

The viscosity of the formulated gel was measured at 25 °C using a cup and bob viscometer (AMETEK Brookfield; USA) with spindle number 5 at the torque rate of 53%.

#### *In-vitro* drug release of the prepared gel

In-vitro drug release of the prepared gel was done using three vertical Franz diffusion cells with nitrocellulose membrane (pore size 0.45 µm and diameter 47 mm) and PBS at pH = 7.4 as the receiver phase at 37 °C and stirring at 100 rpm. Samples were collected at predetermine intervals and replaced with an equal volume of fresh PBS. The concentration of clindamycin and FLZ in the samples was determined using a UV spectrophotometer.

#### Antimicrobial activity of clindamycin-loaded niosomes and niosomal gel

Standard concentrations of clindamycin in water (0.25, 0.125, 0.0625, 0.03125, 0.015625, 0.0078, 0.0039, 0.001953, 0.00097, 0.000488 mg/ml) were prepared. *Staphylococcus aureus* and *Lactobacillus* were cultured in Muller Hinton agar and MRS agar, respectively. They were incubated in an incubator for 24 h; for *Lactobacillus*, the incubation was performed in an anaerobic jar in the incubator (Memmert; Germany). The minimum inhibitory concentration of clindamycin for Staphylococcus aureus and *Lactobacillus* were 0.6 ng/ml and 2 µg/ml, respectively. The diameters of the growth inhibition zone of the standard concentrations were measured using the disk diffusion method. The linear range of the resulting diagrams was identified and a clindamycin concentration within this range was selected as the positive control. The unloaded niosomes as negative control, selected concentrations of clindamycin as the positive control, clindamycin-loaded niosomes (at the same concentration as the positive control), and clindamycin-loaded niosomes gel were evaluated three times using the disk diffusion method [21–23].

#### Antifungal activity of FLZ-SLNs and FLZ-SLNs gel

Standard concentrations of FLZ in water (10, 5, 2.5, 1.25, 0.626 mg/ml) were prepared. *Candida albicans* (*C. albicans*) was cultured in Sabourraud dextrose agar and incubated for 24 h. Measuring the diameters of growth inhibition zones using disk diffusion method revealed an almost linear diagram. The minimum inhibitory concentration of FLZ for *C. albicans* was 0.125 mg/ml. A concentration was selected as the positive control. Unloaded SLNs as the negative control, selected FLZ concentration as the positive control. FLZ-SLNs, and FLZ-SLNs gel were evaluated three times using disk diffusion method [24, 25].

#### Statistical analyses

Quantitative results were expressed as mean ± standard deviation. Statistical differences were analyzed by using ANOVA (*P* < 0.05).

## Results and discussion

### FT-IR

**Fig. 1:**
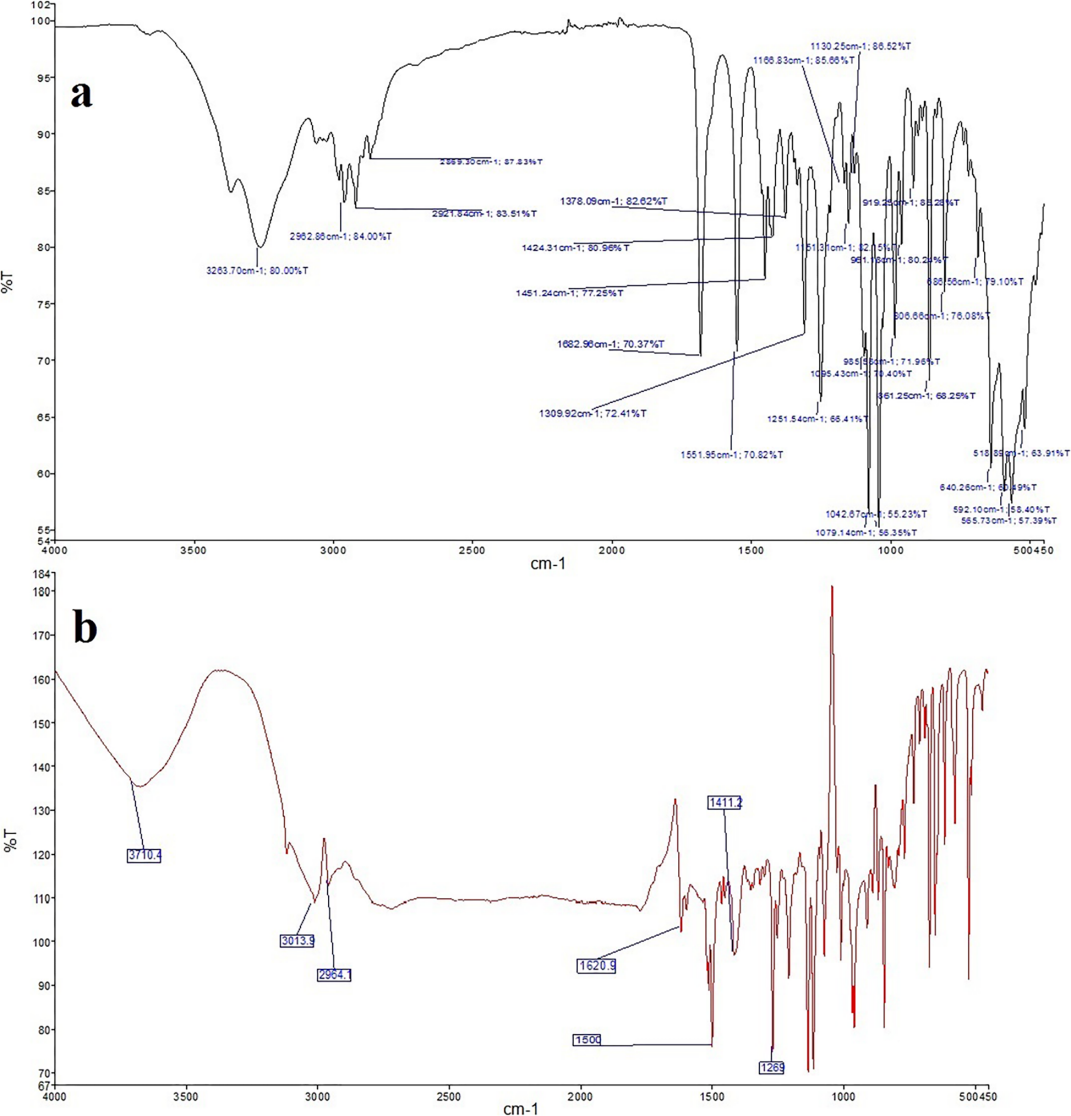
FT-IR spectra: **a** pure clindamycin, **b** pure FLZ

FT-IR spectra of pure clindamycin and FLZ were obtained (Fig. 1) to identify both drugs based on the functional group corresponding to each wavelength of clindamycin and FLZ as shown in Table 1.

**Table 1:** Wavelengths of the functional groups of clindamycin and FLZ in the FT-IR spectra

Functional groups of clindamycin	Wavelength of functional groups of clindamycin	Functional groups of FLZ	Wavelength of functional groups of FLZ
C-O cyclic ether stretching	1151.31/1079.14	C = C stretching of aromatic ring	1620
S-C-H bending	1251.54/1309.92		3013.9
N–C = O stretching of amid carbonyl group	1682.96/1551.95	C-H stretching of aromatic ring	
C–Cl stretching	861.25	CH2 stretching	2964.1
C-N stretching	1451.24		
C–C stretching	1042.67		
C-H stretching	2921.84/2962.86		
O–H stretching	3263.7		

### Calibration curve of clindamycin and FLZ

**Fig. 2:**
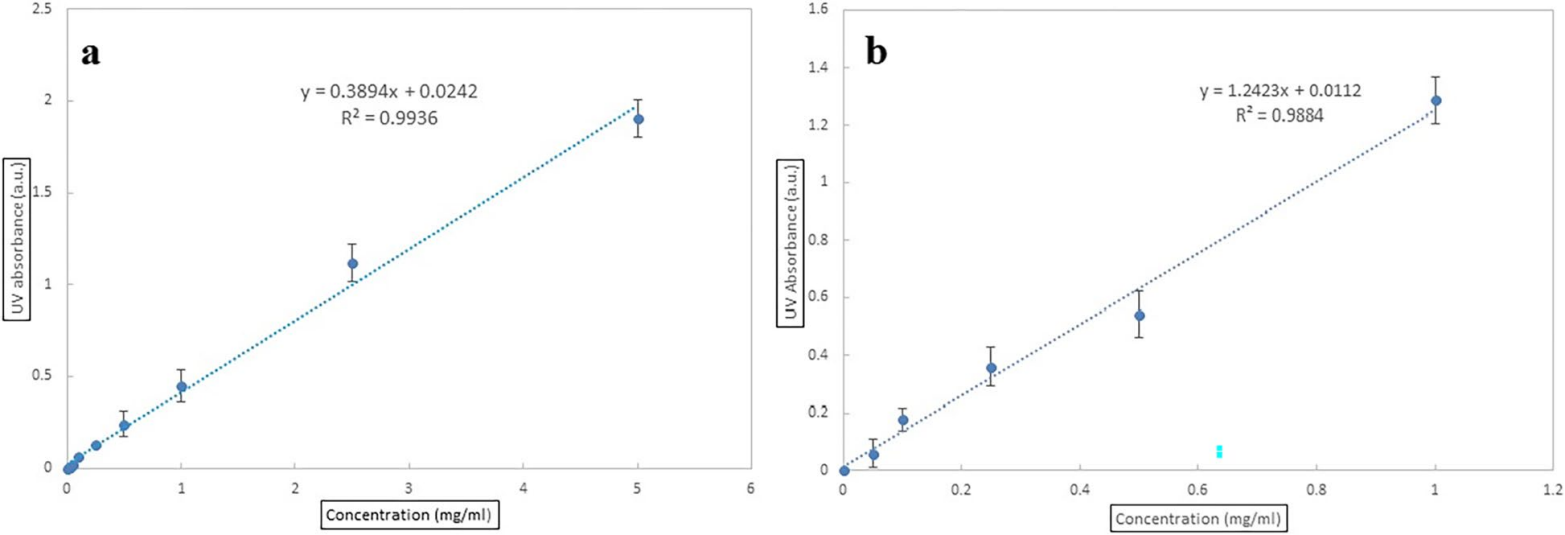
Calibration curve: **a** clindamycin, **b** FLZ

The calibration curves of clindamycin and FLZ were prepared using a UV spectrophotometer at 225 and 253 nm, respectively (Fig. 2). Both calibration curves were almost linear and they were subsequently utilized for other tests, including *in-vitro* drug release and the percentage of entrapment efficacy.

### Differential scanning calorimetry (DSC)

**Fig. 3:**
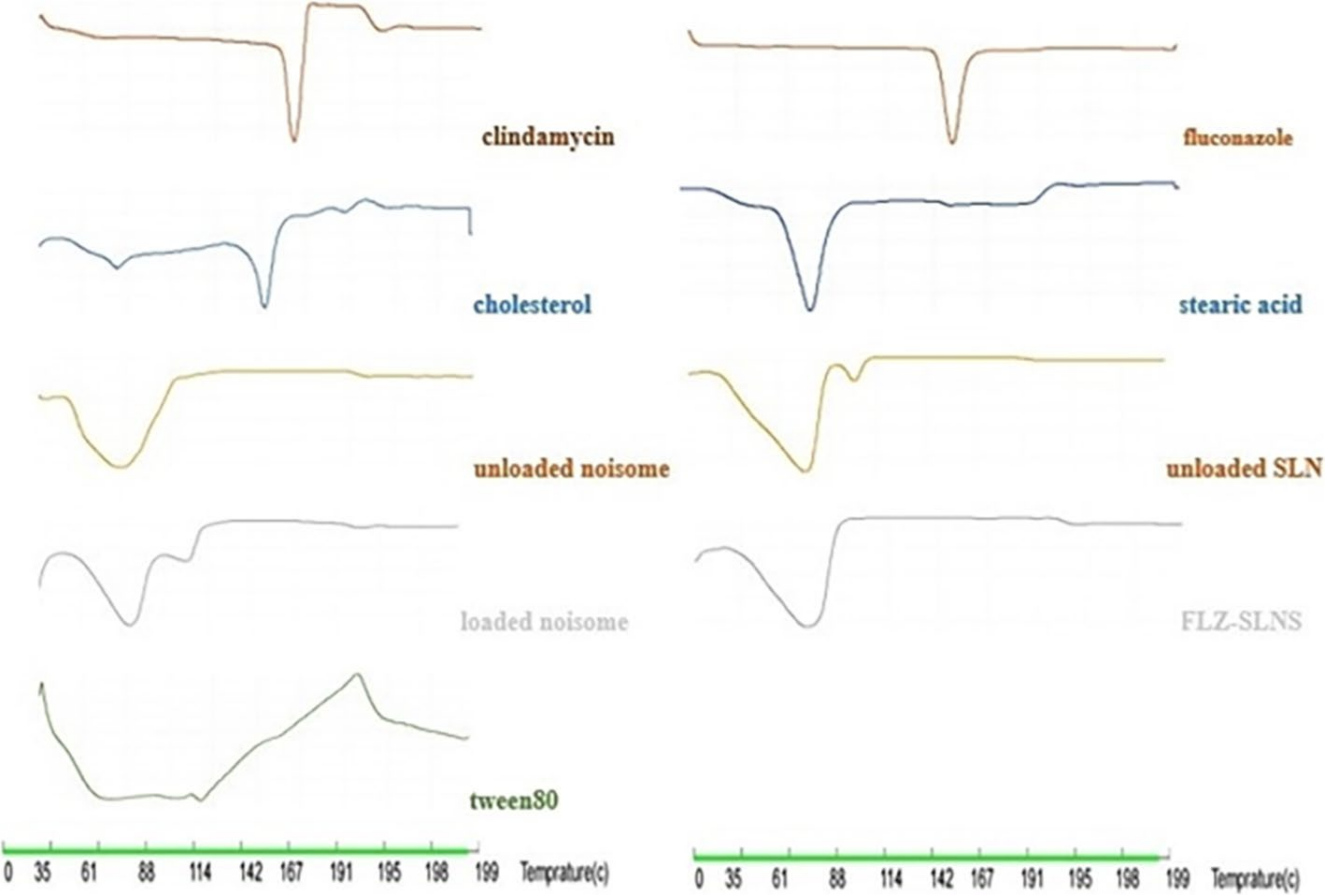
Thermograms of DSC

DSC thermograms were obtained by plotting heat flux against temperature. Thermograms were obtained for pure clindamycin, cholesterol, tween 80, unloaded noisome, clindamycin-loaded niosomes, FLZ, stearic acid, unloaded SLNs, and FLZ-SLNs (Fig. 3). The endothermic and exothermic peaks for each sample are summarized in Table 2. According to the table, endothermic peaks of clindamycin and FLZ are not seen in the diagrams of clindamycin-loaded niosomes and FLZ-SLNs that means both of drugs are successfully entrapped in nanoparticles. In noisome formulation we do not see endothermic peaks of cholesterol and tween 80 because of their participation in the structure and the new peak around 60 °C is due to the formation of lipid bilayers. Also in SLNs formulations endothermic peaks of tween 80 and stearic acid are not seen and the new peak around 65 °C in FLZ-SLNs formulation is due to the formation of the structure.

**Table 2:** Sum of thermograms of DSC descriptions

Samples	Endothermic peak	Exothermic peak
Clindamycin	150 °C (melting point of clindamycin)	——————
Cholesterol	45 °C and 145 °C, the endothermic peak at 45 °C is due to the transformation of cholesterol crystal polymorphs into each other, while the endothermic peak at 145 °C corresponds to the melting point of cholesterol	——————
Tween 80	Broad endothermic peak at 40 °C -100 °C, boiling point of tween 80	190 °C (flash point of tween 80)
Unloaded noisome	60 °C, due to the formation of niosomes. The disappearance of the endothermic peaks of cholesterol and tween 80 indicates their participation in the formulation structure	——————
Clindamycin-loaded niosomes	62 °C, 97 °C, the endothermic peak observed at 62 °C is attributed to the formation of niosomes. The endothermic peak of clindamycin disappeared, indicating successful entrapment of the drug within niosomes. The endothermic peak observed at 97 °C corresponds to solvent evaporation	
FLZ	145 °C, melting point	
Stearic acid	75 °C, stearic acid melting point	
Unloaded SLNs	78 °C and, 105 °C. The endothermic peaks of tween 80 and stearic acid are not seen due to their participation in the formulation structure. The endothermic peak at 105 °C is due to solvent evaporation. The endothermic peak at 78 °C is attributed to stearic acid that did not incorporate into the formation of the SLNs structure	
FLZ-SLNs	65 °C. The endothermic peak of FLZ is not seen, indicating that the drug is successfully entrapped. Nor are the endothermic peaks of tween 80 and stearic acid seen due to their participation in the formulation structure. The endothermic peak at 65 °C corresponds to the formation of the structure	

### Entrapment efficacy

The EE% was 98% ± 0.3 for clindamycin-loaded niosomes and 51% ± 0.7 for FLZ-SLNs. Previous studies have shown that incorporating cholesterol in the niosomes structure increases the loading of water-soluble drugs, such as clindamycin [26]. High EE% of clindamycin loaded niosomes is also because of high water solubility of clindamycin in the core of niosomes. Increasing the proportion of lipid leads to higher loading of fat-soluble drugs like FLZ. Additionally, the use of solid lipids with longer chains and more hydrophobic properties can increase drug loading [17]. In this study, the use of stearic acid with long chains at a ratio of 0.33% w/w resulted in an average loading of FLZ inside the SLNs.

### Particle size and zeta potential

**Fig. 4:**
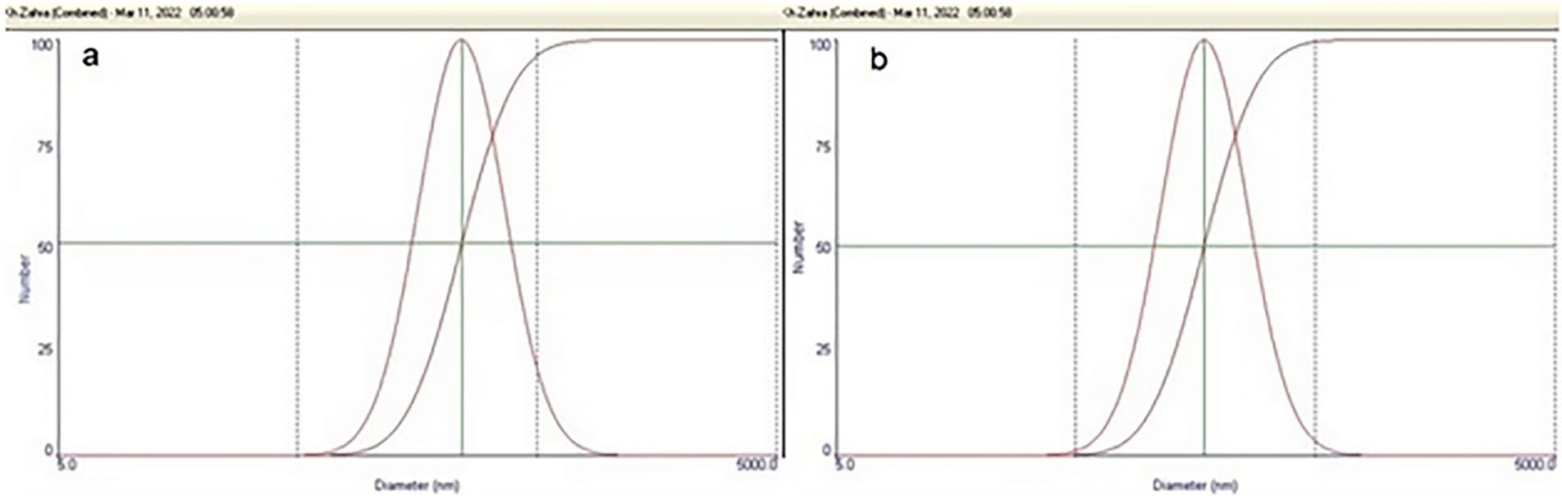
Results of DLS regarding the mean diameters: **a** niosomes, **b** FLZ-SLNs

The mean diameters of loaded niosomes and FLZ-SLNs were 243.03 nm and 171.97 nm, respectively (Fig. 4). The difference in size between these two formulations can be attributed to the use of different manufacturing methods, different concentration of surfactant, and the incorporation of different lipids with varying proportions in their structure [27]. Generally, both formulations are in the nanometer size that leads them reach to the infected cells and then release the loaded drug at the site of infection. One of the reasons of low particle size of SLNs is the low lipid ratio of 0.33%w/w stearic acid. The higher the lipid ratio, the larger the particle size [28]. Also, tween 80 as a surfactant in nanoparticles leads to the larger size in comparison with other surfactants. In this study tween 80 concentration of SLNs formulations (1%w/v) is more than the niosomes formulations (0.95%w/v). Increasing the concentration of surfactant decrease the particle size of nanoparticles [29]. So higher concentration of tween 80 in SLNs formulations is one of the reasons of its lower size.

**Fig. 5:**
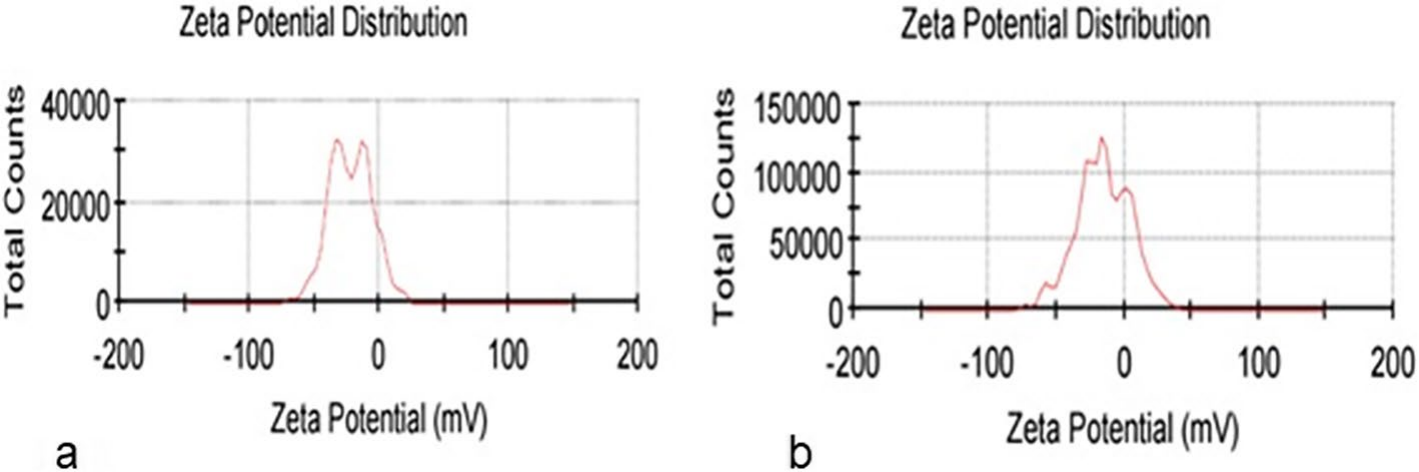
Mean zeta potential: **a** niosomes, **b** FLZ-SLNs

The mean zeta potential of clindamycin-loaded niosomes and FLZ-SLNs were -23.3 and -15, respectively (Fig. 5). Zeta potential refers to the charge at the interface between a solid surface and the surrounding liquid medium. It serves as a reliable index to assess the state of the nanoparticles surface and predict the long-term stability of a colloidal dispersion. Particle diameter profoundly impacts the zeta potential, as small diameter particles are more susceptible to the Brownian motion of the fluid flow and neighboring particles, resulting in a higher absolute value of the effective zeta potential. A negative zeta potential of sufficient magnitude is indicative of stable formulation.

### TEM

**Fig. 6:**
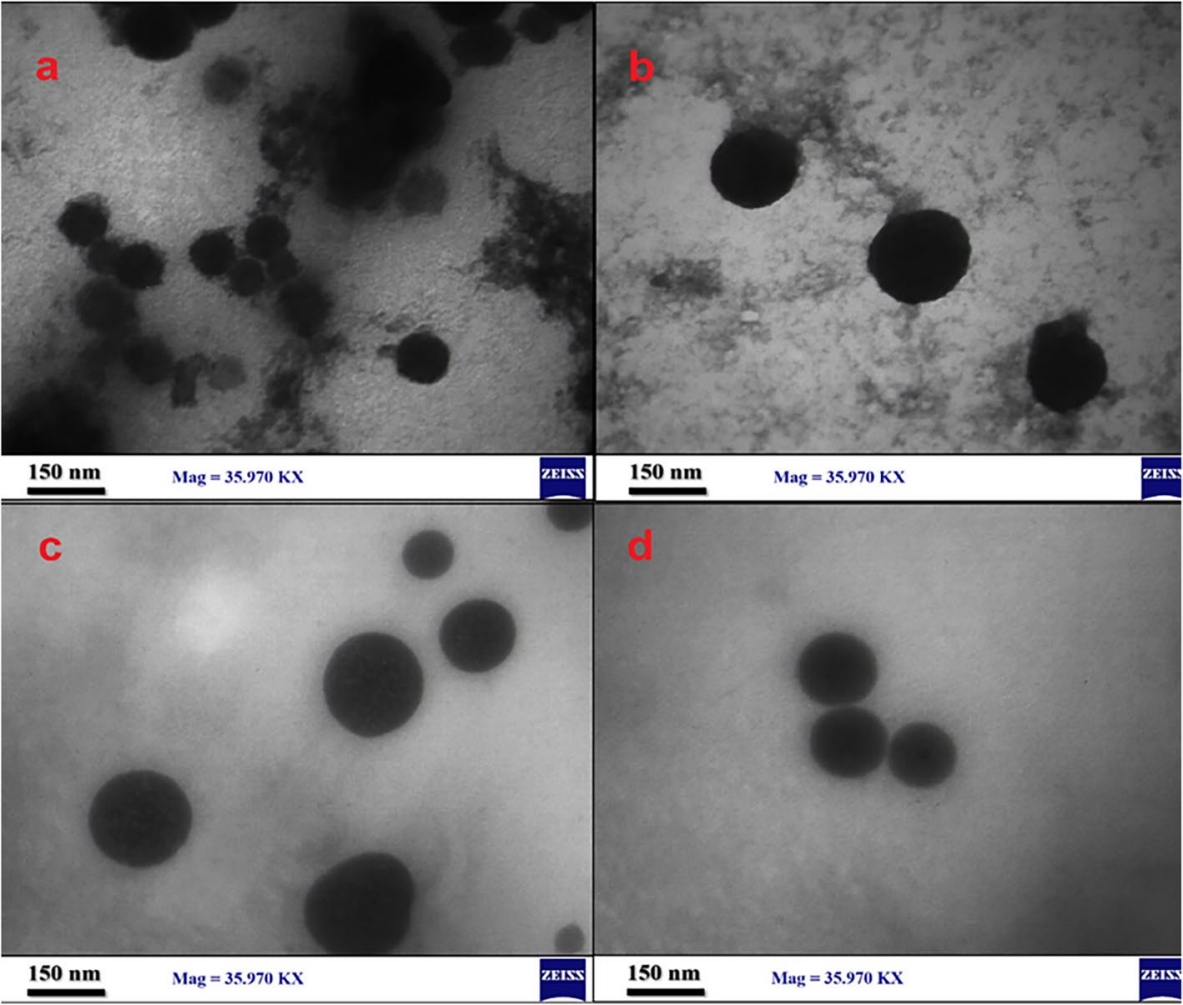
**a** Unloaded niosomes, **b** loaded niosomes, **c** unloaded SLNs, **d** loaded SLNs

The obtained results demonstrated that both formulations of niosomes (with and without clindamycin) and SLNs (with and without FLZ) are spherical and almost monodispersed. Moreover, the entrapped clindamycin and FLZ did not exhibit any signs of crystallization despite their ability to form crystals. Additionally, no particle aggregation was observed (Fig. 6).

### Gelling temperature and viscosity measurement

The two main prerequisites for an effective in-situ gelling system are viscosity and gelling temperature. The formulation should possess the desired viscosity to provide optimal coverage of the affected area upon administration. Furthermore, the formulation must undergo a rapid solution-to-gel transition upon contact with the affected site to ensure its efficacy [20, 30]. The gel under study exhibited a viscosity of 2132 cp at the gelling temperature of 33 °C.

### *In-vitro* drug release from FLZ-SLNs and clindamycin-loaded niosomes and the prepared gel

**Fig. 7:**
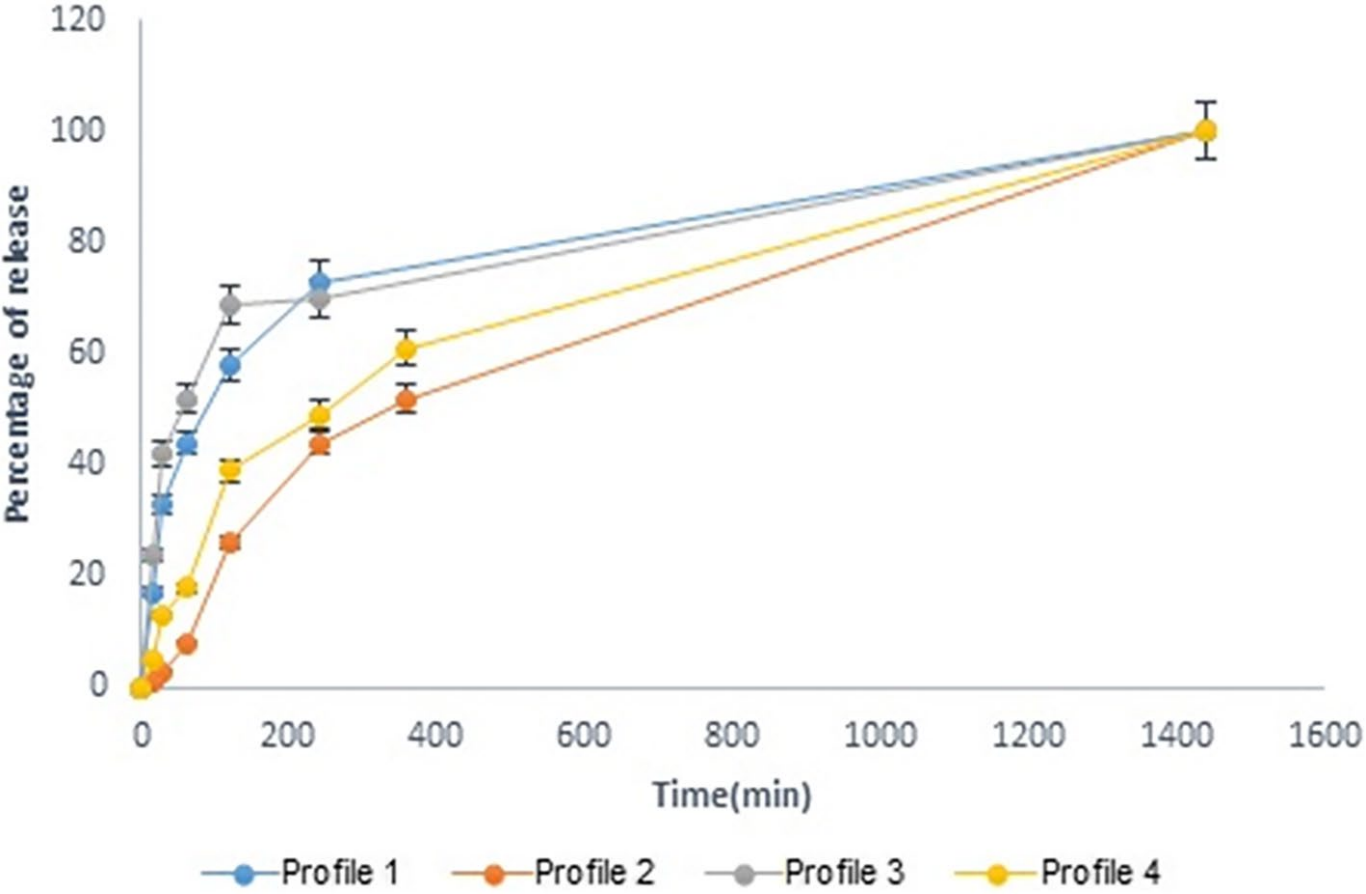
Profile 1: clindamycin-loaded niosomes, Profile 2: clindamycin-loaded niosomes in gel formulation, Profile 3: FLZ-SLNs, Profile 4: FLZ-SLNs in gel formulation

All four formulations showed complete drug release within 24 h, with both niosomes and SLNs displaying rather similar release profile. Niosomes formulation release the drug slower than SLNs formulation. One reason is the higher specific surface area due to the smaller particle size of SLNs nanoparticles that cause faster drug release. Also, high surfactant concentration of SLNs (1%) can cause faster drug release in comparison with niosomes formulation [29]. The release of drugs from the gel formulations was slower than that from niosomes and SLNs, likely due to the presence of a polymer matrix created by poloxamer in the gel formulation (Fig. 7).

### Antimicrobial activity of clindamycin-loaded niosomes and niosomal gel

**Fig. 8:**
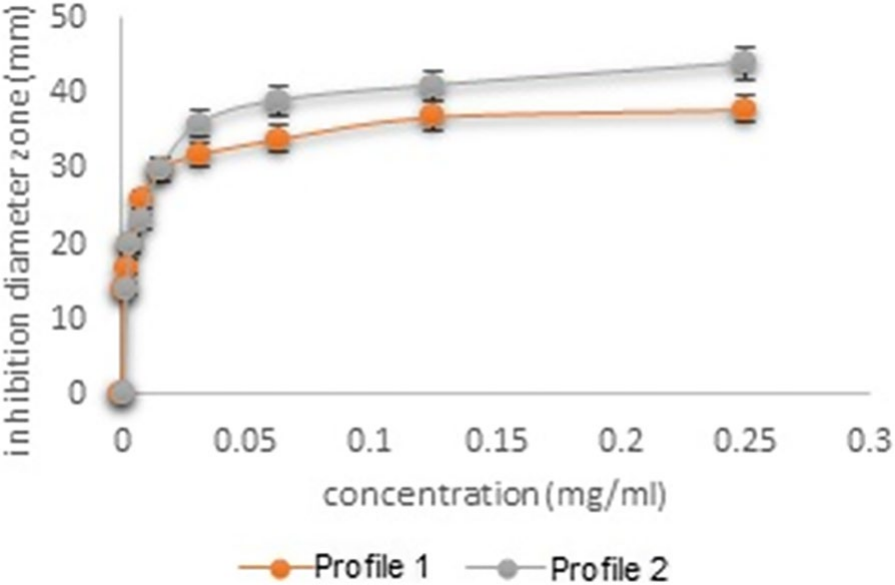
Profile 1: inhibition diameter zone of *S. aureus* versus standard concentration of clindamycin. Profile 2: inhibition diameter zone of *Lactobacillus casei* versus standard concentration of clindamycin

**Fig. 9:**
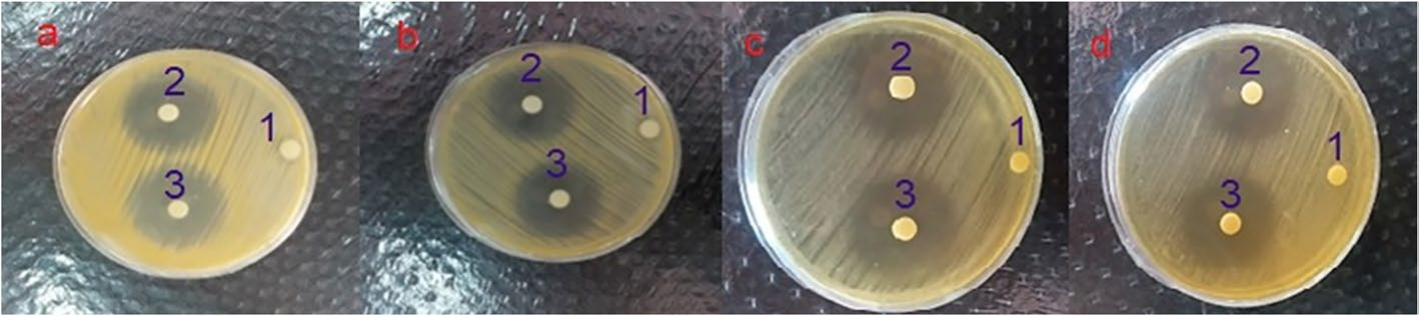
**a** Inhibition diameter zone of *S. aureus*: 1) negative control, 2) positive control, 3) clindamycin-loaded niosomes. **b** Inhibition diameter zone of *S. aureus*: 1) negative control, 2) positive control, 3) niosomal gel. **c** Inhibition diameter zone of *Lactobacillus casei*: 1) negative control, 2) positive control, 3) clindamycin-loaded niosomes. **d** Inhibition diameter zone of *Lactobacillus casei*: 1) negative control, 2) positive control, 3) niosomal gel

Figure 8 displays diagrams of the inhibition diameter zone of *S. aureus* and *Lactobacillus casei* versus the standard concentration of clindamycin in water. The inhibition diameter zones of all formulations against *S. aureus* and *Lactobacillus casei* are summarized in Table 3 and Fig. 9. Measuring the inhibition zone of diameter expressed the sensitivity of the bacteria, which defines the bacteria as resistant (≤ 9 mm), moderately sensitive (10–11 mm), or sensitive (≥ 12 mm) to the antibiotics [31]. Statistical analysis of the results indicated that clindamycin was released from the noisome formulations including clindamycin-loaded niosomes and niosomal gel within 24 h and significantly affected both *S. aureus* and *Lactobacillus casei*, in comparison with the positive control.

**Table 3:** Inhibition diameter zone of *S.aureus* and *Lactobacillus casei* against different formulations (mm)

Formulation	Microorganism	Inhibition diameter of formulation	Inhibition diameter of positive control	Inhibition diameter of negative control	*P* value
Clindamycin-loaded niosomes	*S.aureus*	29 ± 1	32 ± 1	0	0.94
Clindamycin-loaded niosomes in gel	*S.aureus*	30 ± 1	31 ± 1	0	0.97
Clindamycin-loaded niosomes	*Lactobacillus casei*	28 ± 1	30 ± 1	0	0.96
Clindamycin-loaded niosomes in gel	*Lactobacillus casei*	29 ± 1	31 ± 1	0	0.88

### Antifungal activity of FLZ-SLNs and FLZ-SLNs gel

**Fig. 10:**
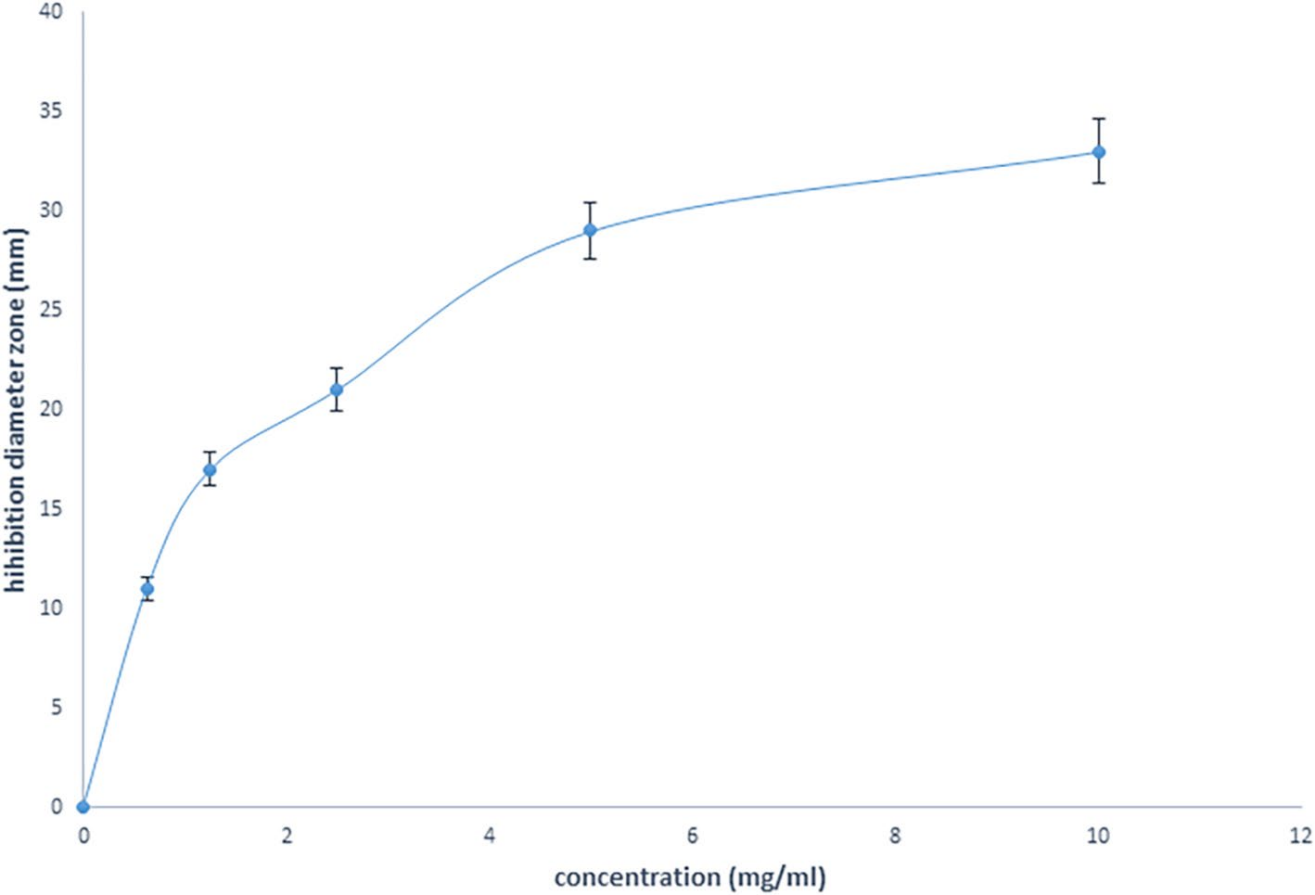
Diagram of the inhibition diameter zone of *Candida albicans* versus standard concentration of fluconazole

**Fig. 11:**
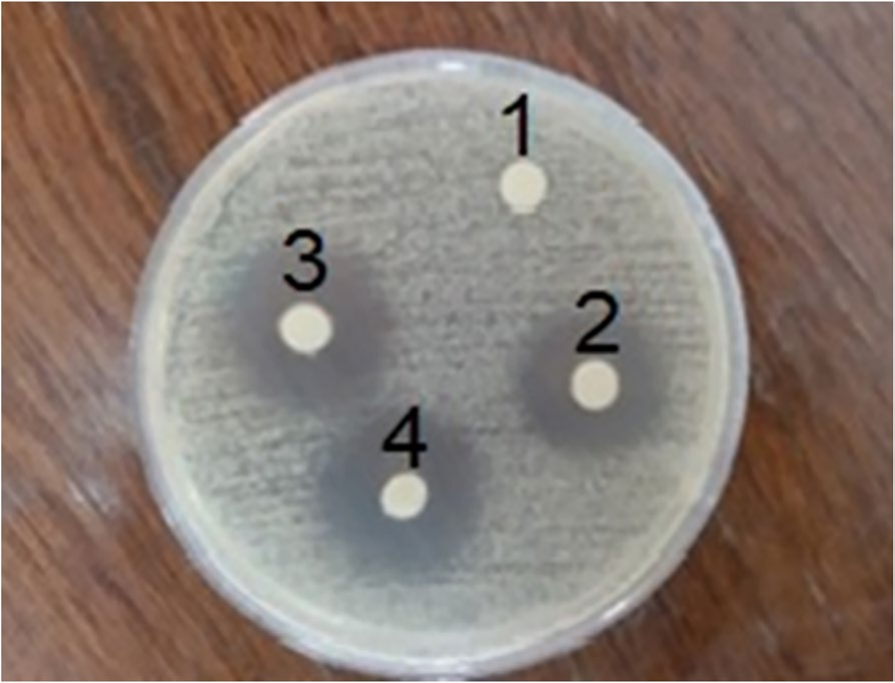
Inhibition diameter zone of *Candida albicans*: 1) negative control, 2) positive control, 3) FLZ-SLNs, and 4) FLZ-SLNs gel

Figure 10 illustrates the diagram of the inhibition diameter zone of *Candida albicans* versus standard concentration of FLZ in water. Figure 11 and Table 4 show the inhibition diameter zones for all formulations. Interpretation of the inhibition zone of diameter expressed the sensitivity of the fungi, which defines them as resistant (≤ 14 mm), moderately sensitive (15–18 mm), or sensitive (≥ 19 mm) to the fluconazole [32]. Statistical analysis of the results showed that both formulations had significant effects in comparison with the positive control, as evidenced by the *P* values.

**Table 4:** Inhibition diameter zone of *Candida albicans* versus different formulations (mm)

Formulation	Microorganism	Inhibition diameter of formulation	Inhibition diameter of positive control	Inhibition diameter of negative control	*P* value
FLZ-SLNs	*Candida albicans*	20 ± 1	22 ± 1	0	0.93
FLZ-SLNs gel	*Candida albicans*	20 ± 1	24 ± 1	0	0.92

## Conclusions

The mean particle size range of the prepared niosomes and SLNs was in the nanometer range, and their entrapment efficiency were acceptable. The thermosensitive gel transitioned from a sol to a gel state at 33 °C, and the release profiles of both gel formulations showed 60% release after 7 h and complete drug release within 24 h. The thermosensitive gel of niosomes loaded with clindamycin and SLNs loaded with FZL had a similar effect to the positive control on typical oral cavity bacteria such as *S. aureus Lactobacillus casei* and typical oral cavity fungi such as *Candida albicans*, respectively, in vitro. The results showed these formulations can be administered as injectable gels in prefilled syringe. At room temperature, they are in solution form at room temperature, but upon injection into the periodontal pockets, they transform into mucoadhesive gels. These gels exhibit sustained drug release for 24 h and effectively eradicate pathogens. Therefore, these formulations can be proposed for in vivo studies.

## Data Availability

The datasets generated and/or analyzed during the current study are available from the corresponding author on reasonable request.

## Abbreviations

SLNs: Solid Lipid Nanoparticles
FZL: Fluconazole
DSC: Differential Scanning Calorimetry
DLS: Dynamic Light Scattering
TEM: Transmission Electron Microscopy
EE: Entrapment Efficiency
